# Supplementary material for: Catalytic oxidation upcycling of polyethylene terephthalate to commodity carboxylic acids
Source: Nat Commun. 2024 Dec 30;15:10732. doi: 10.1038/s41467-024-54822-w (PMC11685807; doi:10.1038/s41467-024-54822-w)
Supplement: Supplementary file 1 — Supplementary Information [file 41467_2024_54822_MOESM1_ESM.pdf]

Supplementary Information

Catalytic Oxidation Upcycling of Polyethylene Terephthalate to Commodity Carboxylic Acids

Qinghai Chen,[1,#] Hao Yan,[1,#,*], Kai Zhao[1], Shuai Wang[1], Dongrui Zhang[1], Yaqian Li[1], Rong Fan[1], Jie Li[1], Xin Zhou[2], Xiaobo Chen[1], Yibin Liu[1], Xiang Feng[1,*], De Chen,[3,*] Chaohe Yang[1]

[1]State Key Laboratory of Heavy Oil Processing, China University of Petroleum (East China), Qingdao 266580, China

[2]College of Chemistry and Chemical Engineering, Ocean University of China, Qingdao, Shandong 266100, China

[3]Department of Chemical Engineering, Norwegian University of Science and Technology, Trondheim 7491, Norway

# Hao Yan, Qinghai Chen contributed equally to this work.

Corresponding author

Correspondence to: Hao Yan (haoyan@upc.edu.cn); Chen De (de.chen@ntnu.no); Xiang Feng

(xiangfeng@upc.edu.cn).

Table of contents

Ⅰ. Supplementary Figures....4

Supplementary Figure 1....4
Supplementary Figure 2....5
Supplementary Figure 3....6
Supplementary Figure 4....7
Supplementary Figure 5....8
Supplementary Figure 6....9
Supplementary Figure 7....10
Supplementary Figure 8....11
Supplementary Figure 9....12
Supplementary Figure 10....13
Supplementary Figure 11....14
Supplementary Figure 12....15
Supplementary Figure 13....16
Supplementary Figure 14....17
Supplementary Figure 15....18
Supplementary Figure 16....19
Supplementary Figure 17....20
Supplementary Figure 18....21
Supplementary Figure 19....22
Supplementary Figure. 22....25
Supplementary Figure 23....26
Supplementary Figure 24....27
Supplementary Figure 25....28

II. Supplementary Tables....29

Supplementary Table 1....29
Supplementary Table 2....31
Supplementary Table 3....32
Supplementary Table 4....33
Supplementary Table 5....34
Supplementary Table 6....35
Supplementary Table 7....36
Supplementary Table 8....37
Supplementary Table 9....38

Supplementary Table 10 39

Ⅲ. Supplementary Methods 40

1.Materials 40

2.Reaction kinetics calculation 40

3.Life cycle assessment 42

I. Supplementary Figures

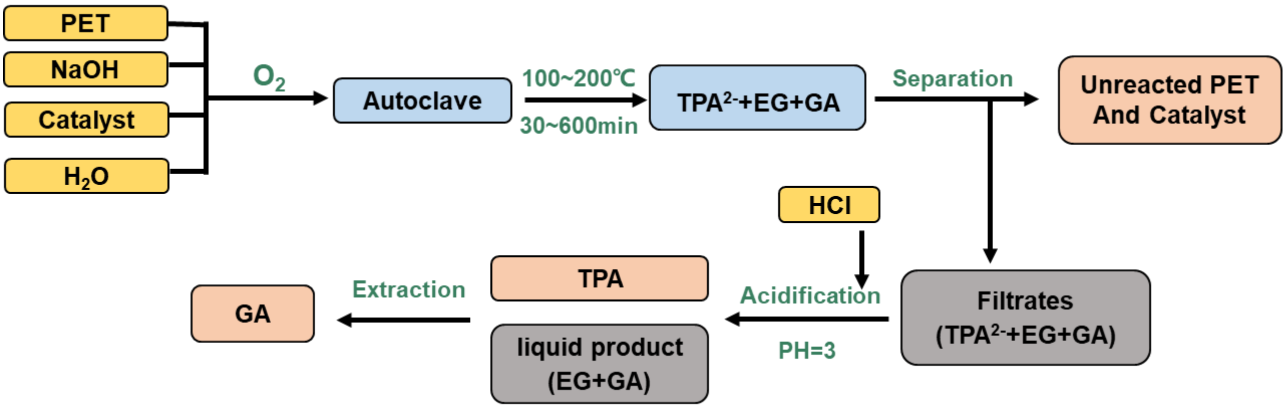

Supplementary Figure 1. PET one-pot process for the production of ethanoic acid under oxygen atmosphere and the process of product separation

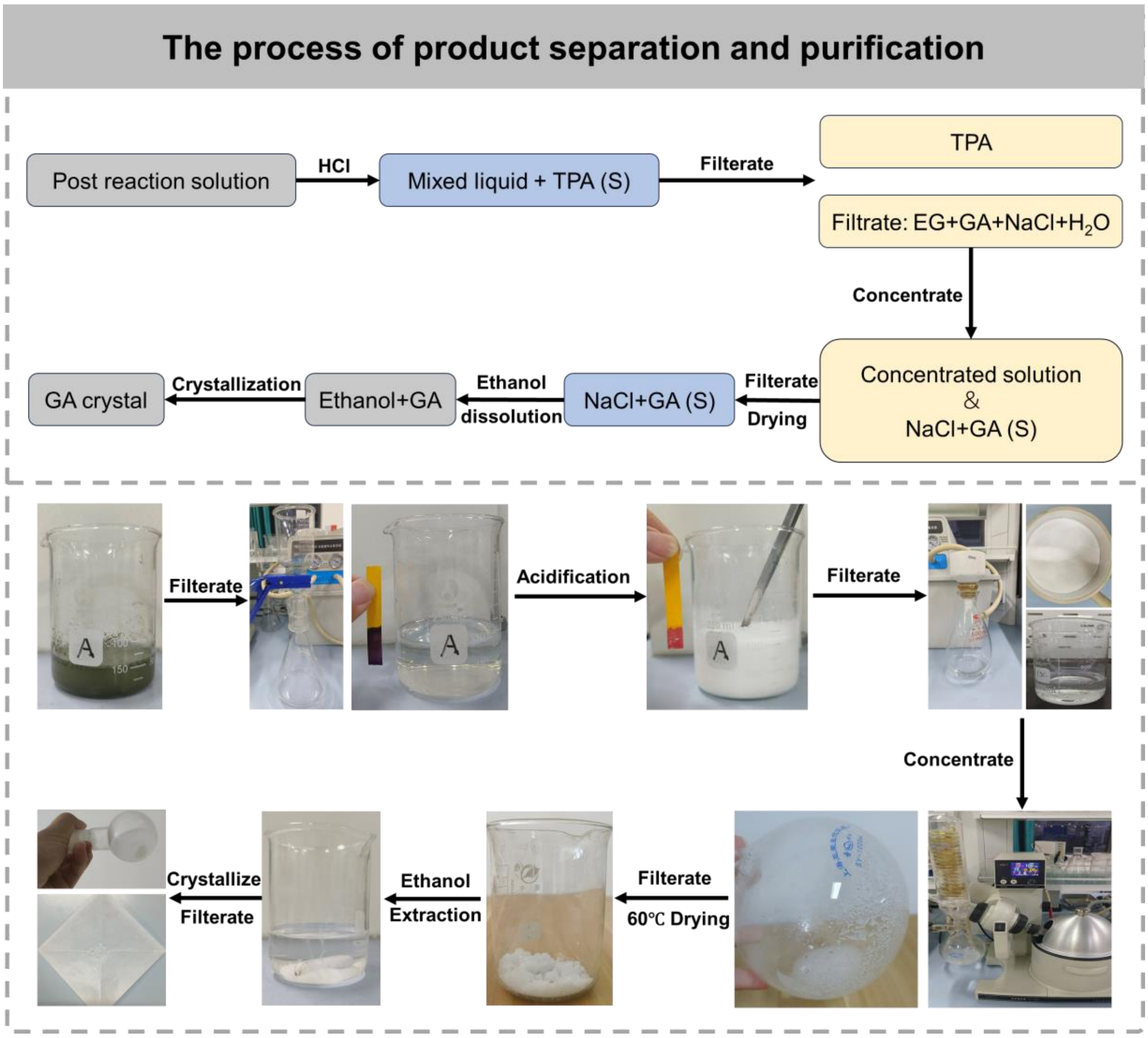

Supplementary Figure 2. The process of product separation and purification.

Separation and purification of products: Firstly, the reaction mixture is filtered to separate the catalyst and unreacted PET. Subsequently, hydrochloric acid (2M HCl) is added to the filtrate for acidification, adjusting the pH to 2~3 to precipitate TPA. After filtering out the formed TPA precipitate, an aqueous solution containing NaCl, ethylene glycol (EG), and glycolic acid (GA) is obtained. The filtrate is then concentrated, allowing glycolic acid and sodium chloride to gradually crystallize and precipitate as water evaporates. After it is concentrated into a certain extent, filtering the concentrated solution can separate ethylene glycol (Liquid at room temperature) from the solid product. Due to the good solubility of glycolic acid in ethanol while NaCl is almost insoluble in ethanol, glycolic acid and NaCl were separated by ethanol extraction. Finally, glycolic acid is crystallized out in ethanol, and after filtration and drying, glycolic acid crystals are obtained, while ethanol is recycled as the mother liquor.

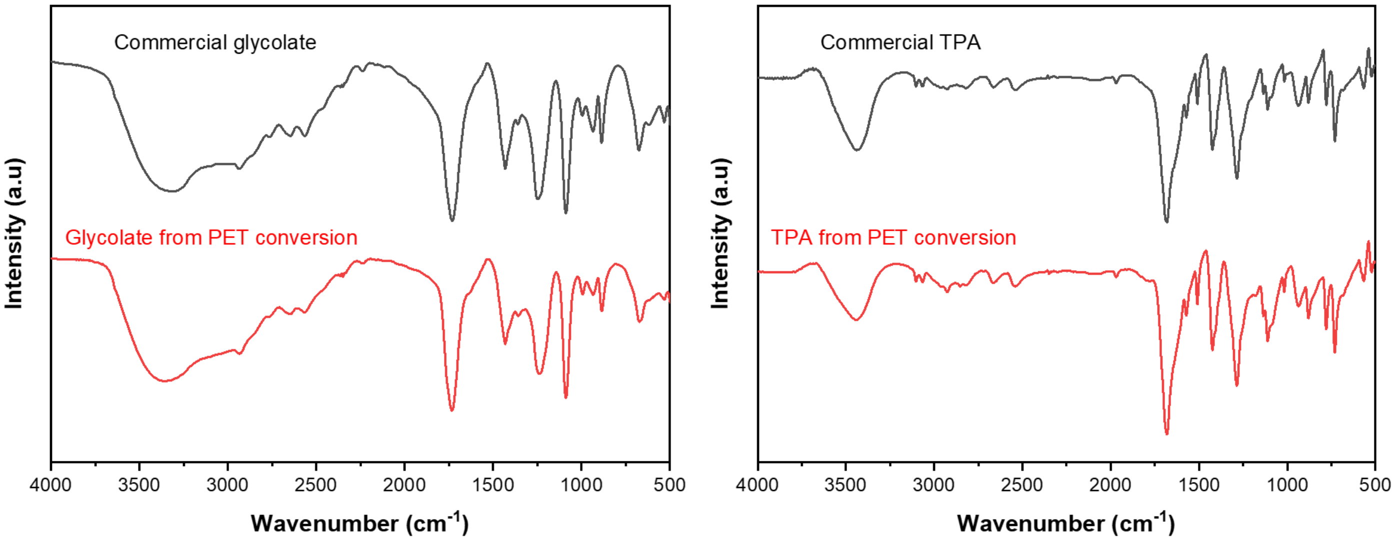

Supplementary Figure 3. FT-IR spectra of the commercial and get from PET conversion of glycolate and TPA.

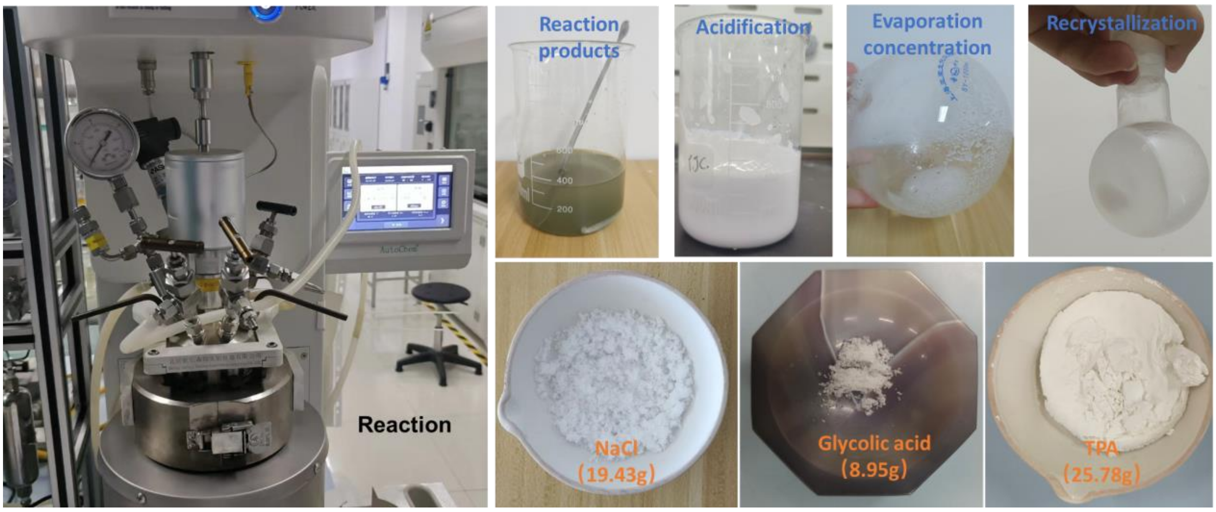

Supplementary Figure 4. The reaction and separation process of 30g PET.

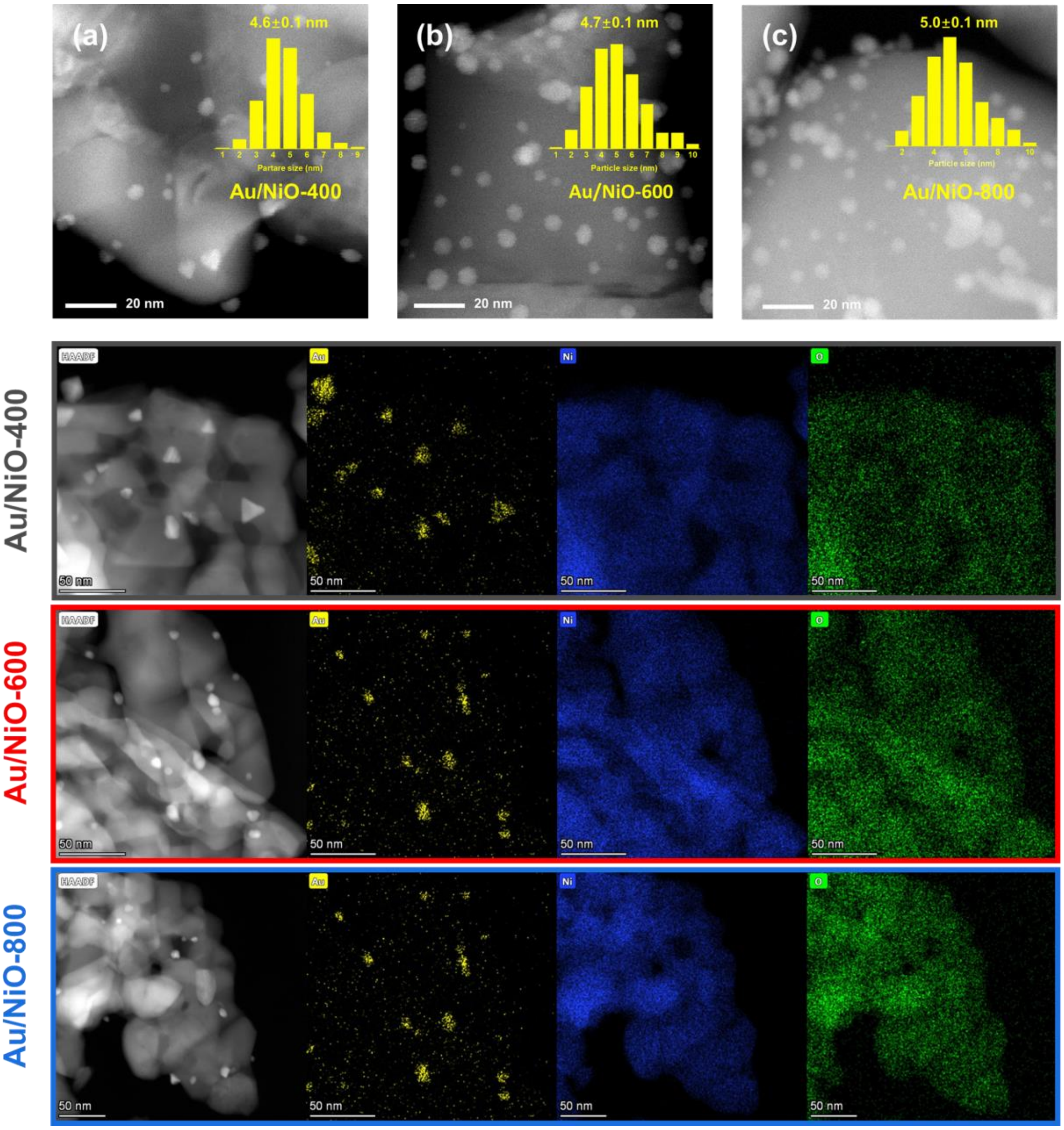

Supplementary Figure 5. TEM images and EDX mapping (Ni, O, Au) of the Au/NiO_2-x catalysts.

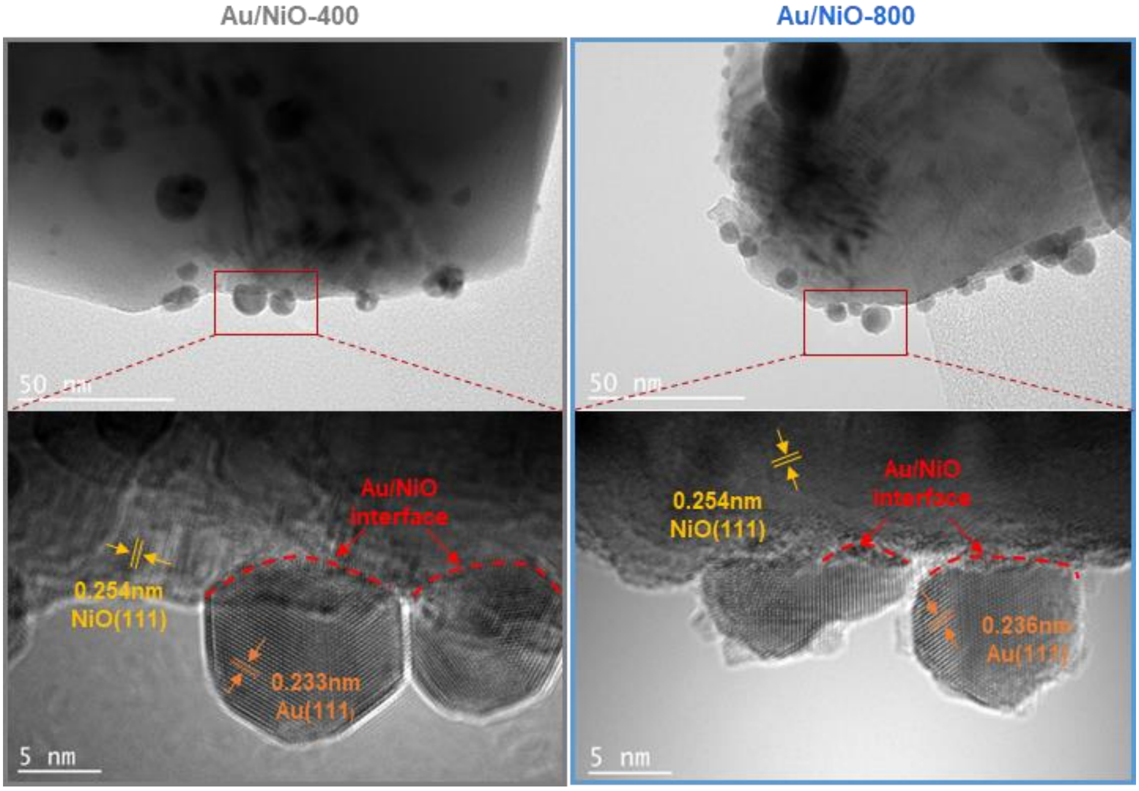

Supplementary Figure 6. HR-TEM images of Au/NiO-400 and Au/NiO-800.

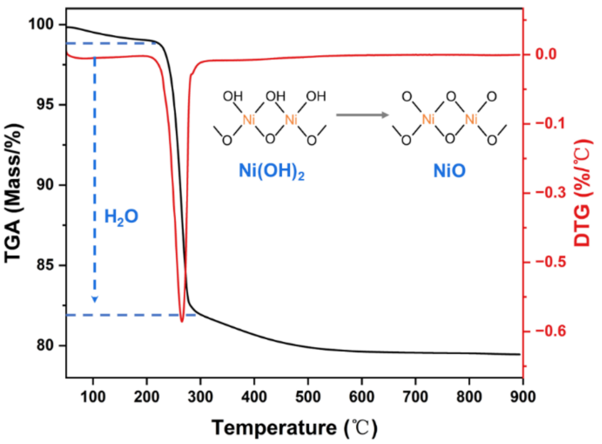

Supplementary Figure 7. TG-DTG curves of uncalcined $Ni(OH)_2$ support.

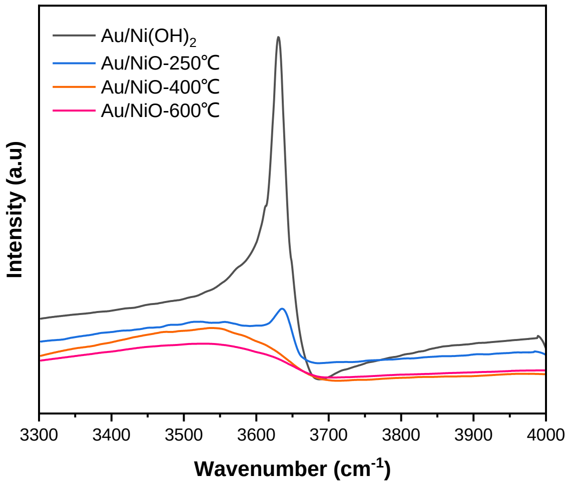

Supplementary Figure 8. Qualitative comparison of the number of hydroxyl groups on the Au/NiO-X surface by FTIR.

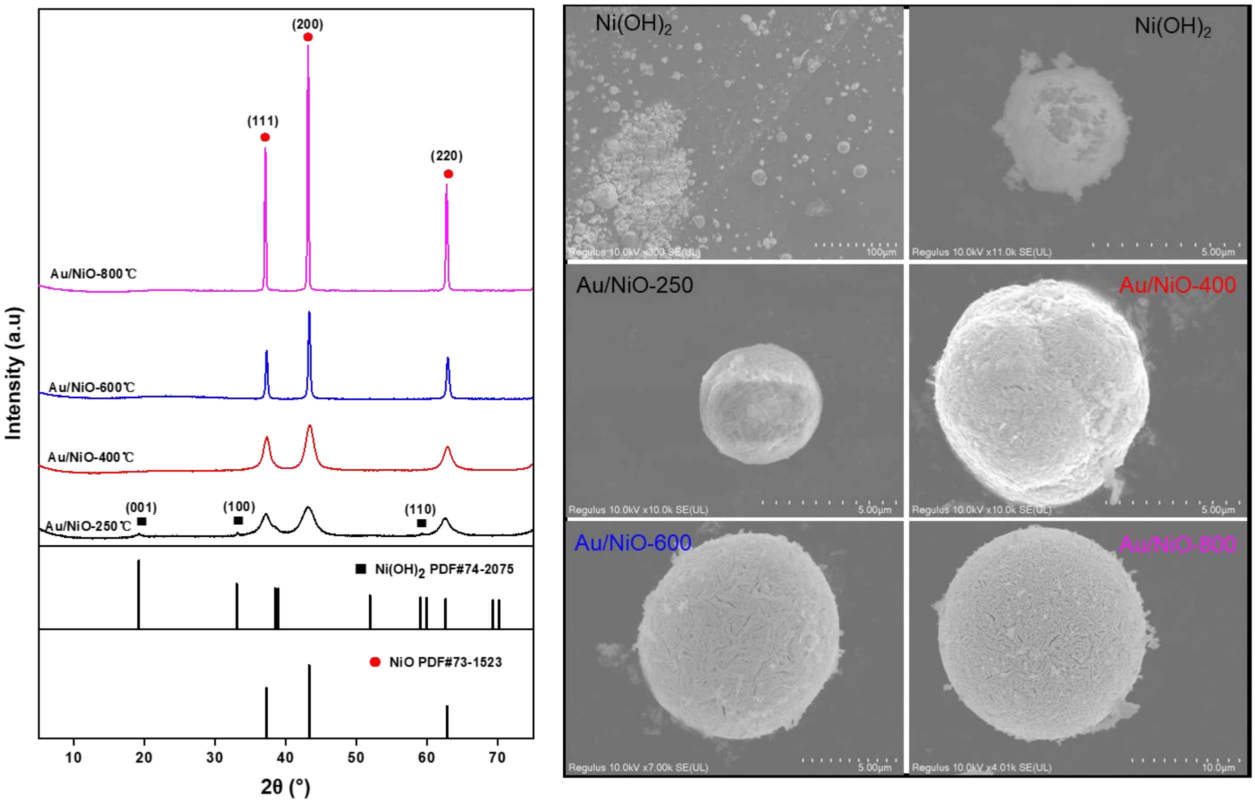

Supplementary Figure 9. XRD patterns of catalysts and SEM images of Au/NiO-x.

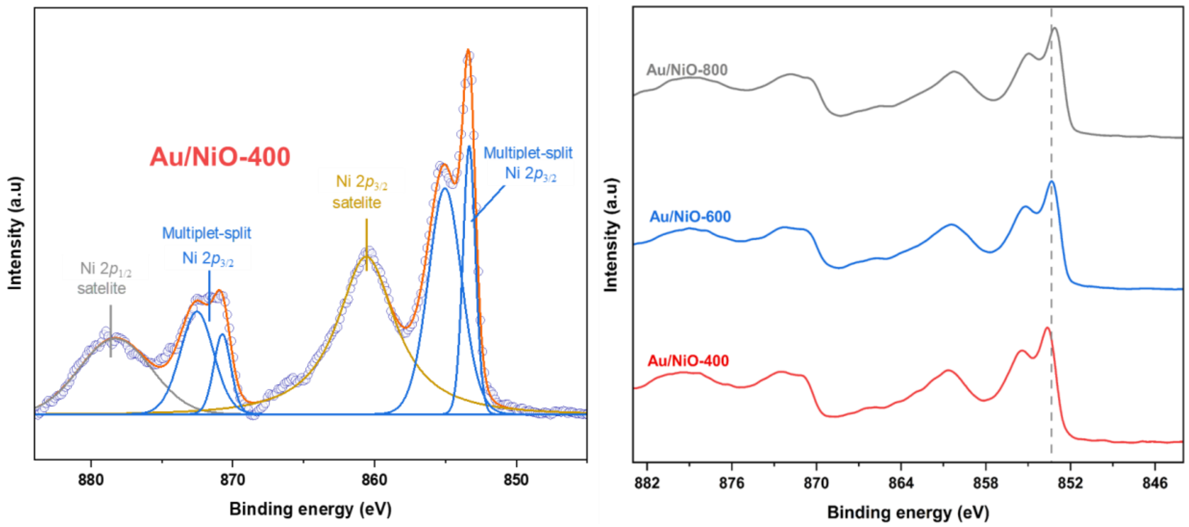

Supplementary Figure 10. Typical characteristic spectral peaks of NiO (Ni 2*p*) and Ni 2*p* XPS spectra of Au/NiO-x.

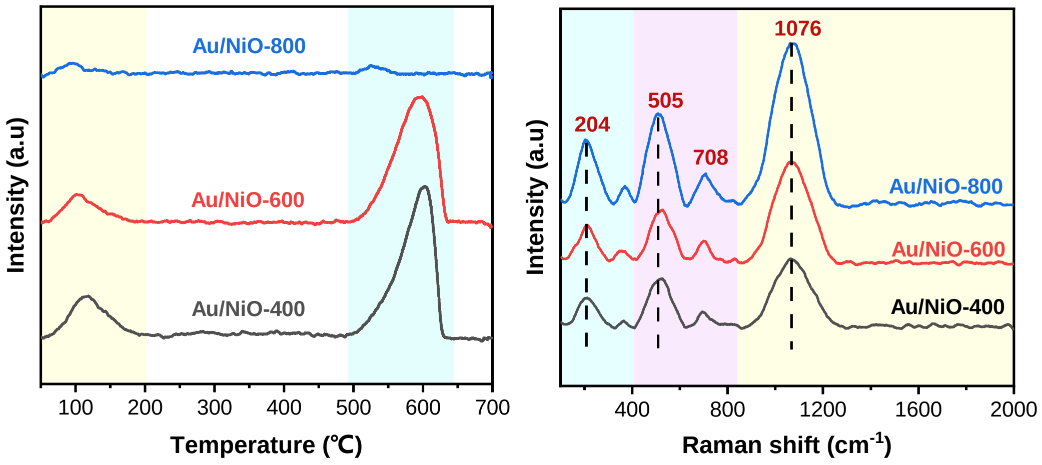

Supplementary Figure 11. O_2-TPD and Raman spectra of Au/NiO-X catalysts.

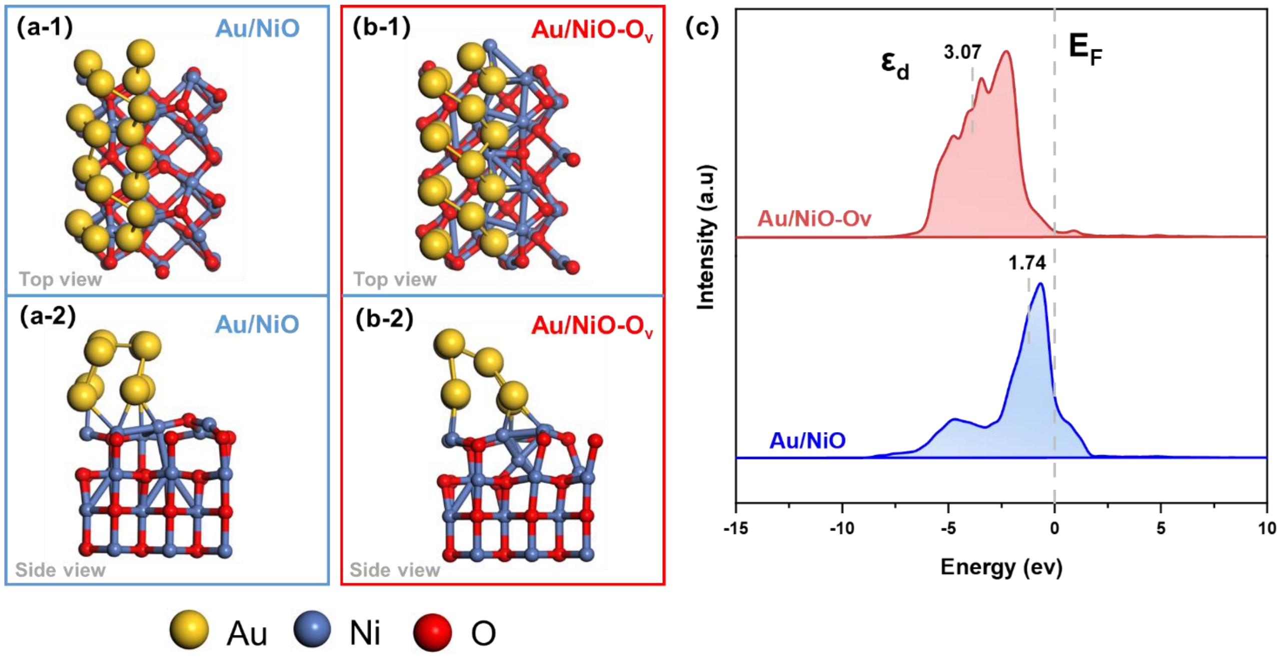

Supplementary Figure 12. DFT calculation models of (a) the Au/NiO and (b) Au/NiO-Ov; (c) Partial density of states (PDOS) of Au/NiO and (b) Au/NiO-Ov.

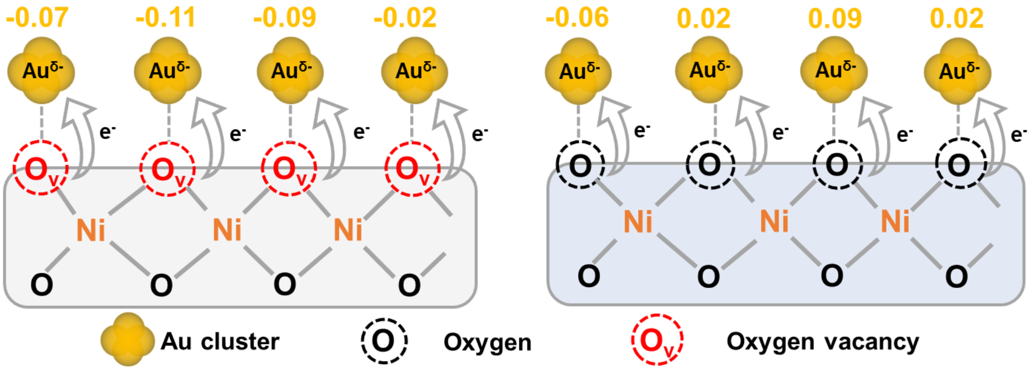

Supplementary Figure 13. Schematic diagram of the Mulliken charge (|e|) distribution for the Au (111) on Au/NiO-Ov model (left) and saturation coordination Au/NiO model (right).

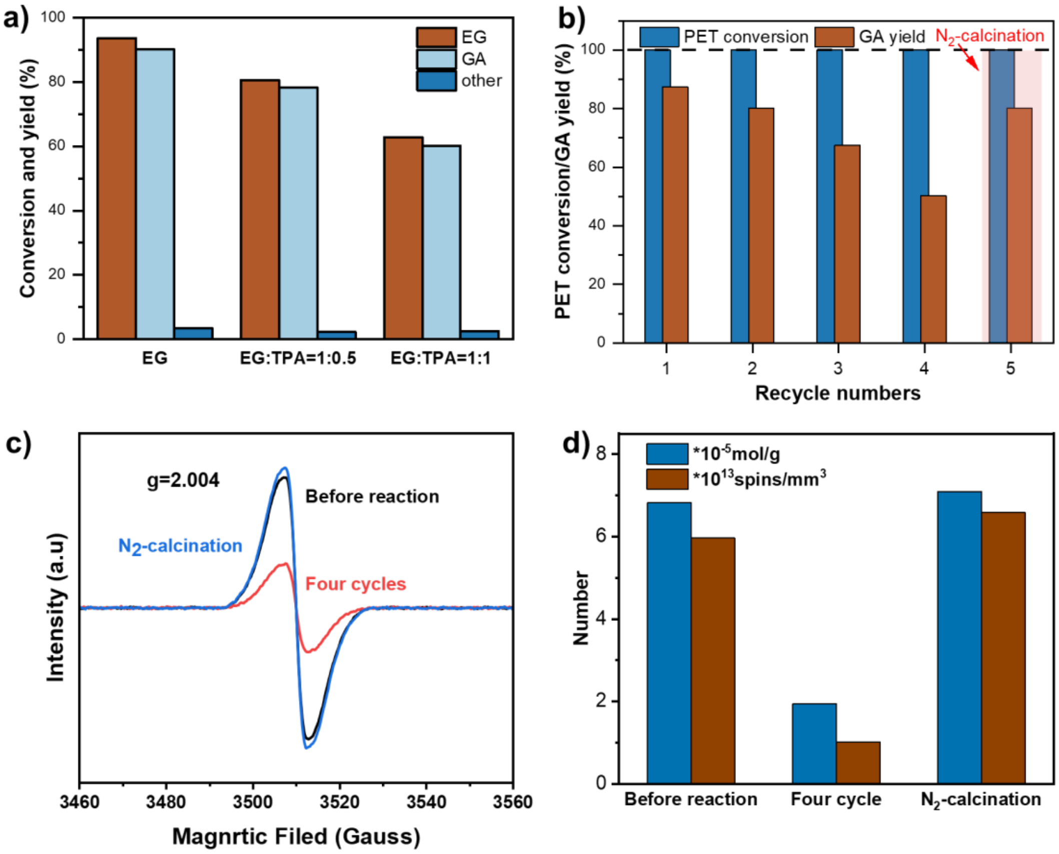

Supplementary Figure 14. a) The catalytic effect of Au/NiO-400 in mixed solutions with different ratios of ethylene glycol and terephthalic acid. (20ml 0.1M ethyene glycol solution, 0.1g catalyst, O.4g NaOH, 1MPa O_2, 80°C, 10min); b) Catalytic stability of the Au/NiO-400 under multiple cycle test conditions (1g PET, 20 ml H_2O, 0.8g NaOH, 0.1g catalyst, 130°C, 3h); c) O_2-TPD spectrum of catalyst at different cycle numbers; d) EPR spectra of catalysts under different cycle numbers.

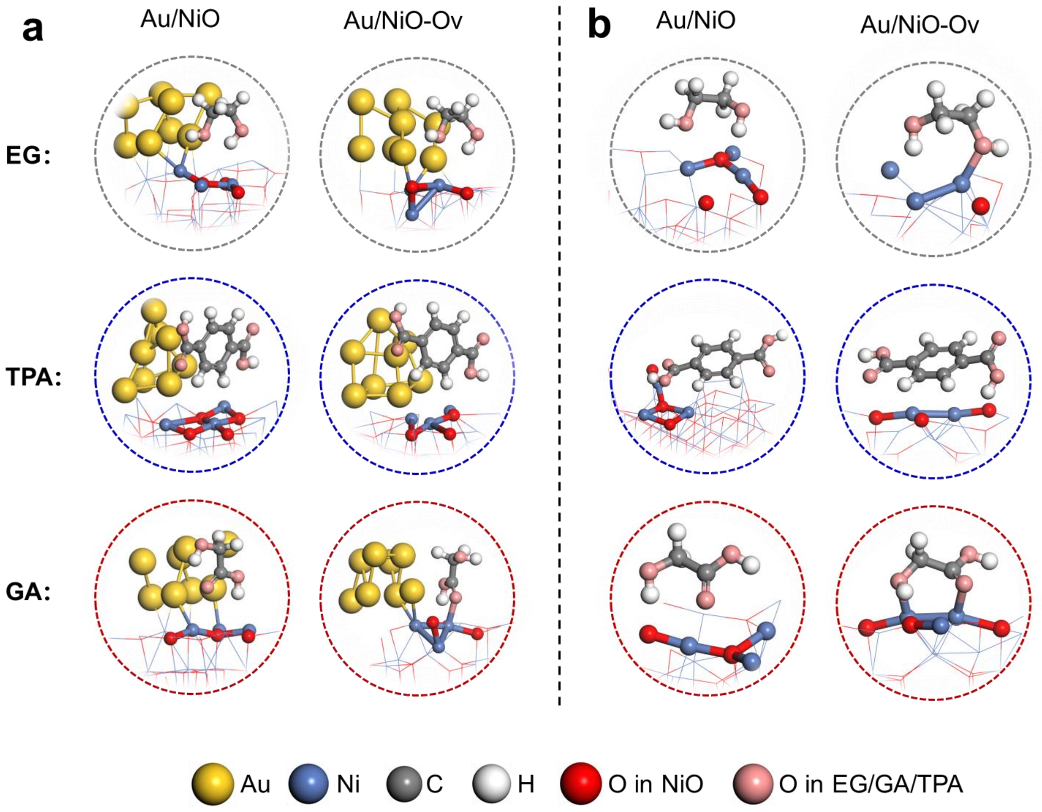

Supplementary Figure 15. Ethylene glycol, terephthalic acid and glycolic acid were adsorbed at the Au/NiO interface (a) and on the NiO carrier surface (b).

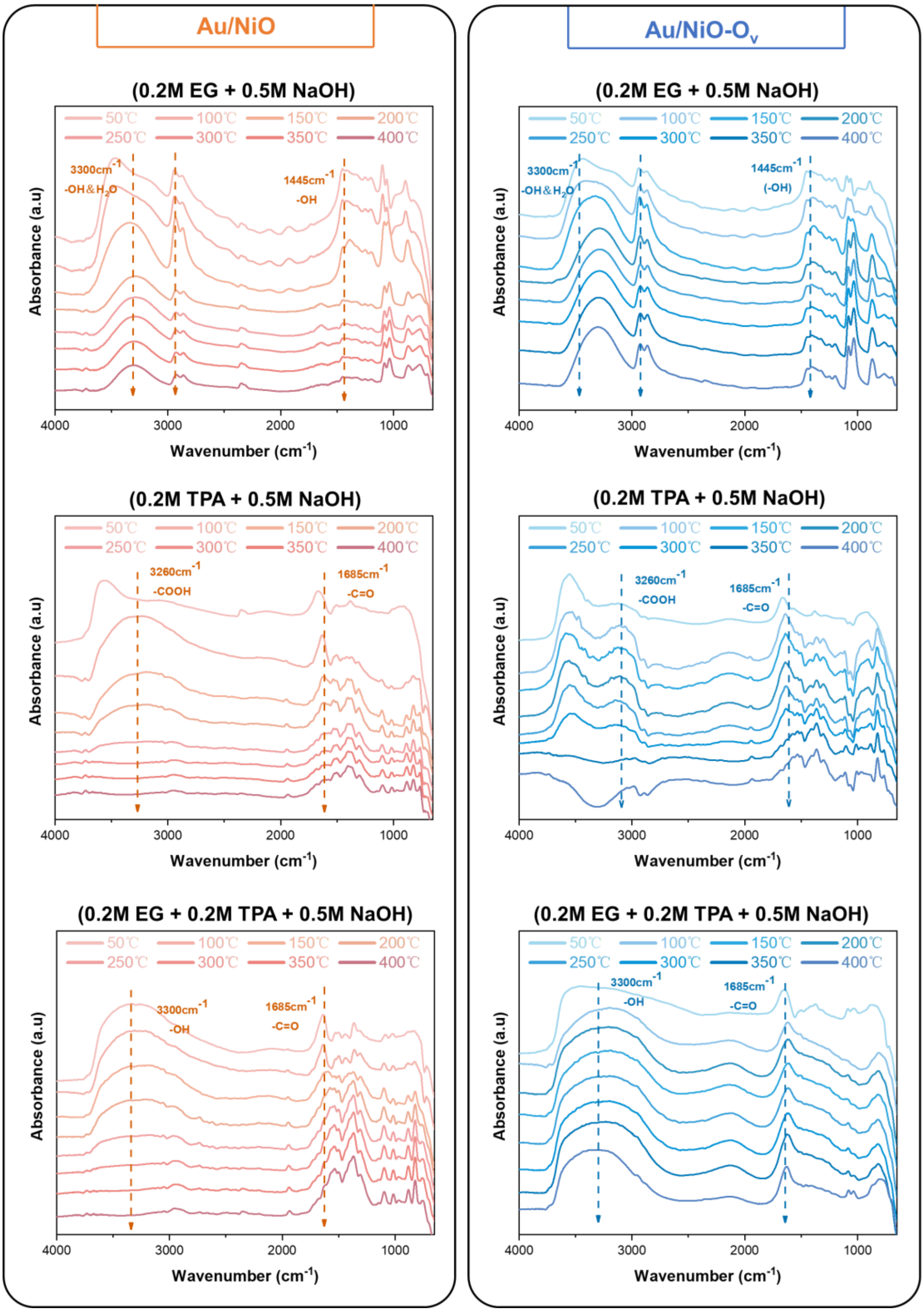

Supplementary Figure 16. In-situ IR spectra of different substrates adsorbed on catalyst surfaces.

Detailed testing process of In-situ IR: The fourier transform infrared spectrum was measured on the Thermo Scientific Nicolet iS50 FTIR. The samples were pretreated by 50mL/min of N_2 at 200°C for 1h. After the sample cools to 30°C, scan and record the sample background. Then add 50ul of the corresponding solution to the in-situ cell. Set the heating program and scan the corresponding sample infrared spectrum under 25ml/min N_2 after reaching the corresponding temperature.

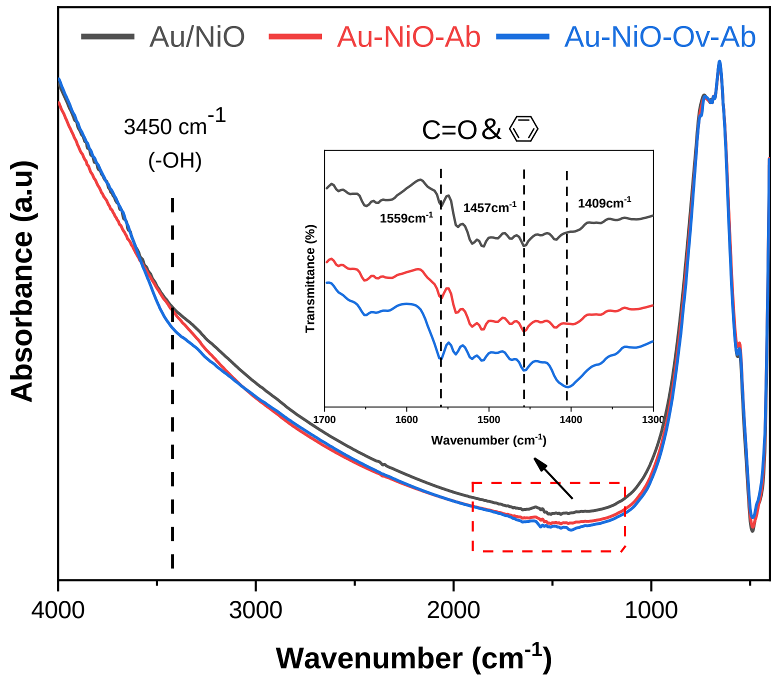

Supplementary Figure 17. FTIR spectra of adsorption of EG and TPA on catalyst surfaces.

Detailed testing process of FTIR: Take 10ml of mixed solution (0.2M TPA, 0.2M EG, 0.5M NaOH) and 0.5g of catalyst (Au/NiO or Au/NiO-O_v), stir at room temperature for 4 hours, and then freeze dry the filtered catalyst. Press 0.1g KBr tablets and scan the spectrum as background. Subsequently, 0.01g of dried catalyst and 0.1g of KBr were mixed evenly and pressed into tablets. The spectra were scanned 64 times with a resolution of 4cm^{-1}.

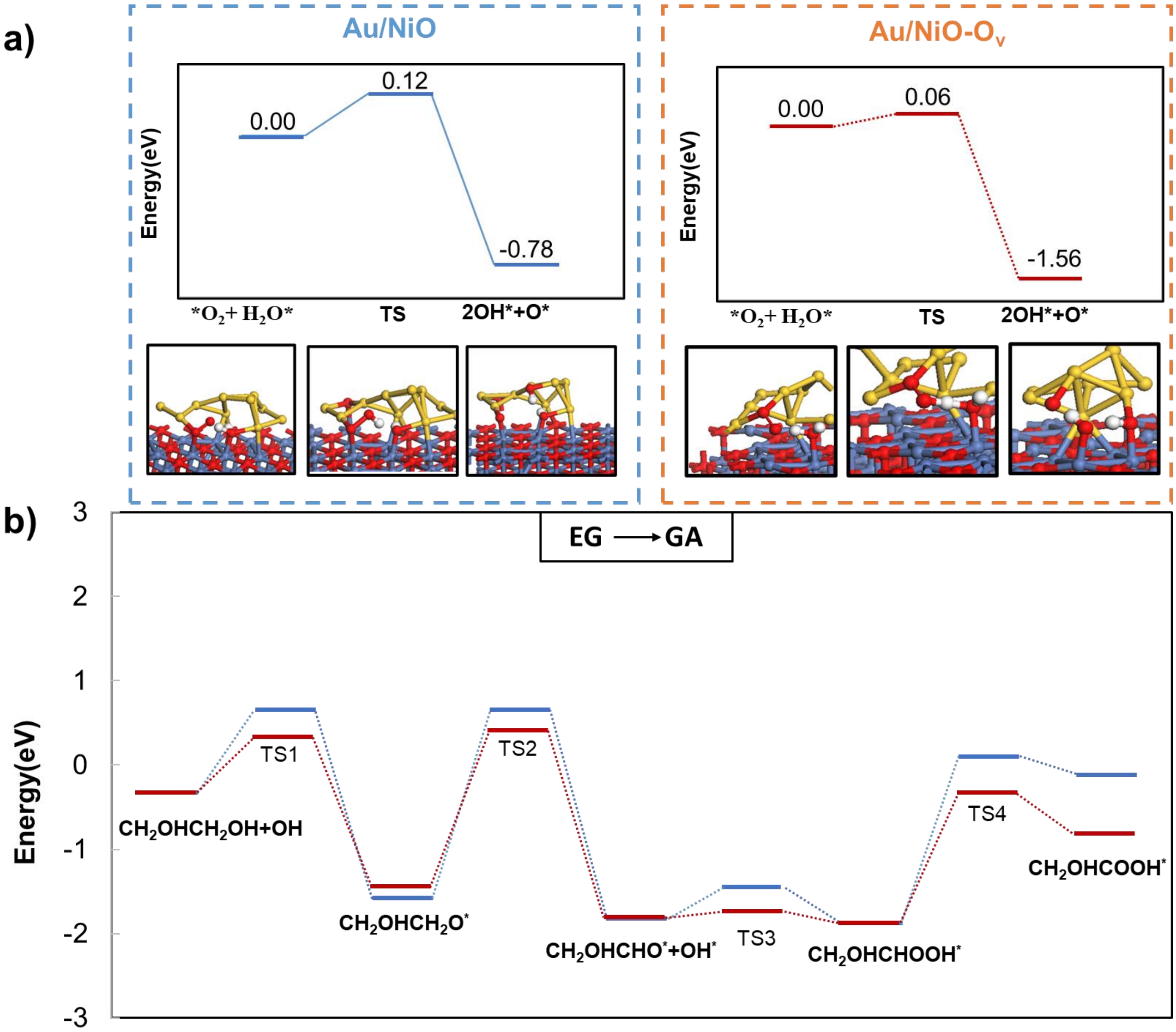

Supplementary Figure 18. a: Activation of oxygen on Au/NiO and Au/NiO-O_v and their respective free energy diagrams;b: Free energy diagrams for the oxidation of ethylene glycol to glycolic acid.

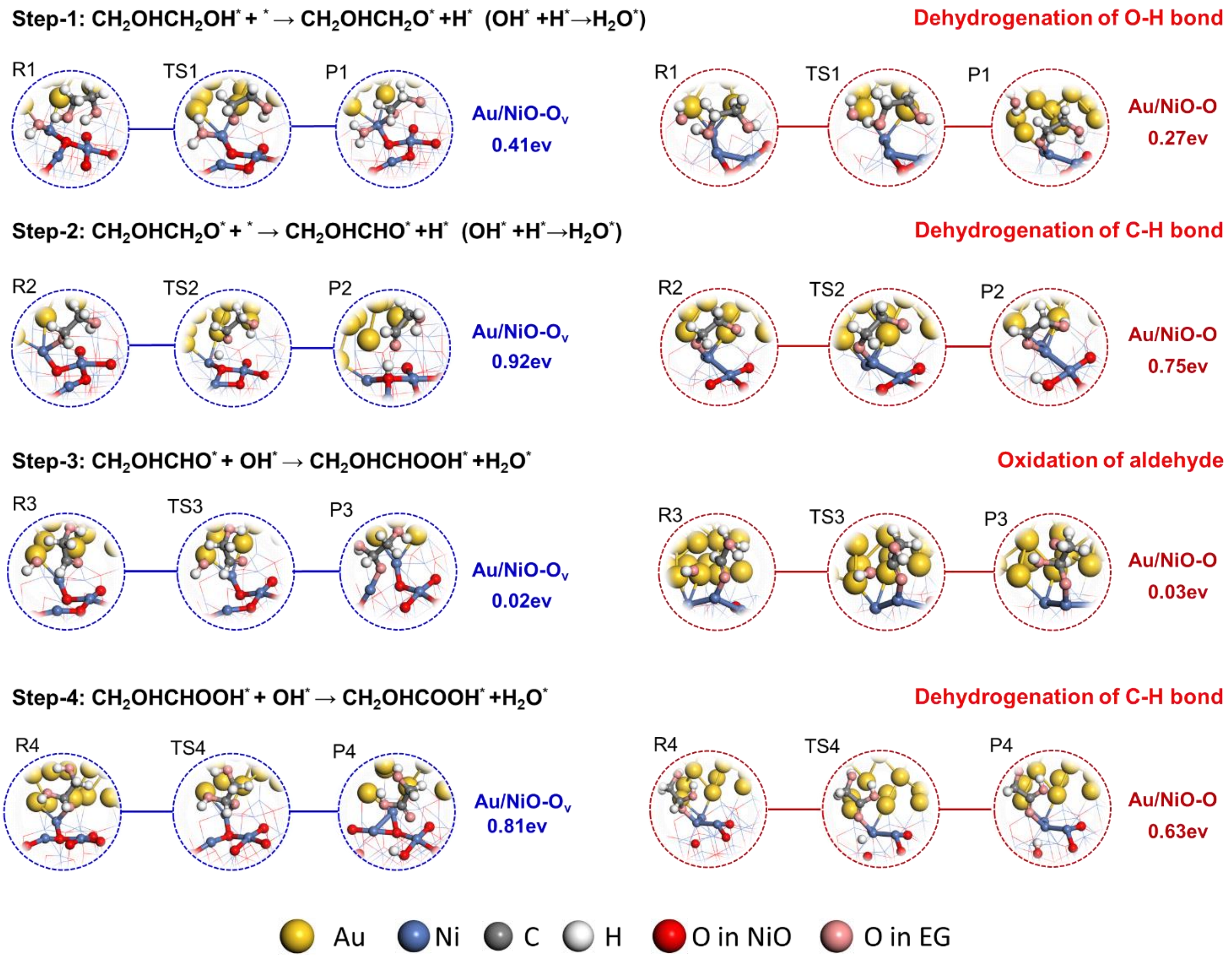

Supplementary Figure 19. Configuration diagrams of reactants, transition states, and products on the Au/NiO-O_v [blue] and Au/NiO [red] (Step-1 to Step-4).

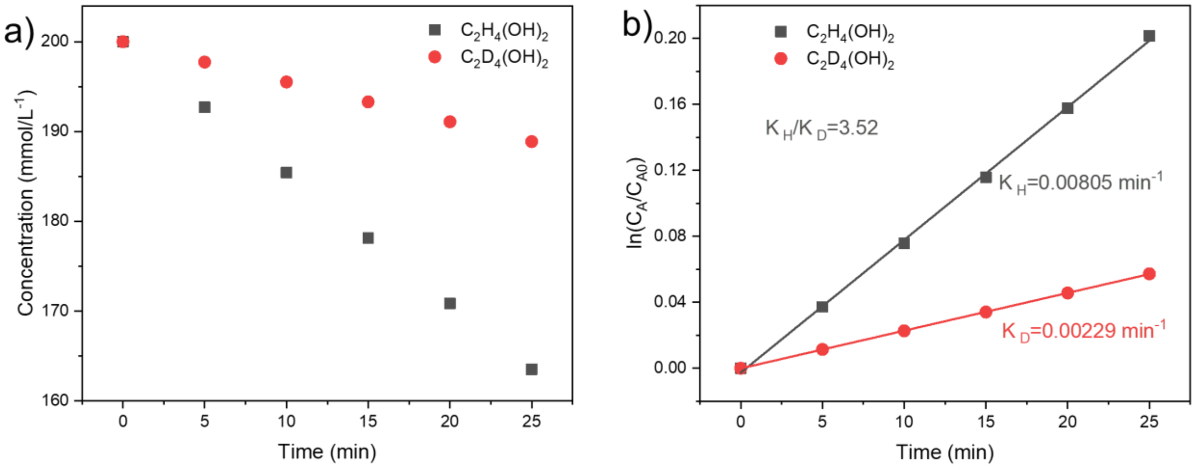

Supplementary Figure. 20. a) Reactant concentration as a function of reaction time and b) kinetic isotope effect in oxidation of $C_2H_4(OH)_2$ and $C_2D_4(OH)_2$.

Reaction conditions: 20mL 0.2M glycol , 50mg catalyst, 1Mpa O_2, 80^oC.

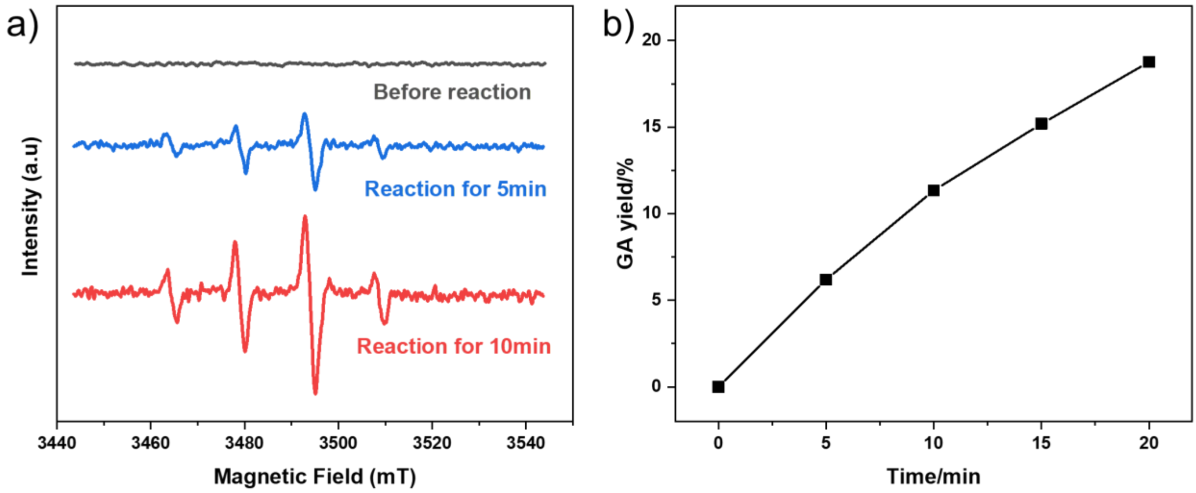

Supplementary Figure. 21. a) In-situ EPR spectra with free radical trapping agent (DMPO) for the oxidation of glycerol on Au/NiO-O_v (Reaction condition: 20ml 0.15M glycolaldehyde dimer, 0.8g NaOH, 0.1g Au/NiO-O_v 100°C); b) Yield of gthanol acid at different reaction times.

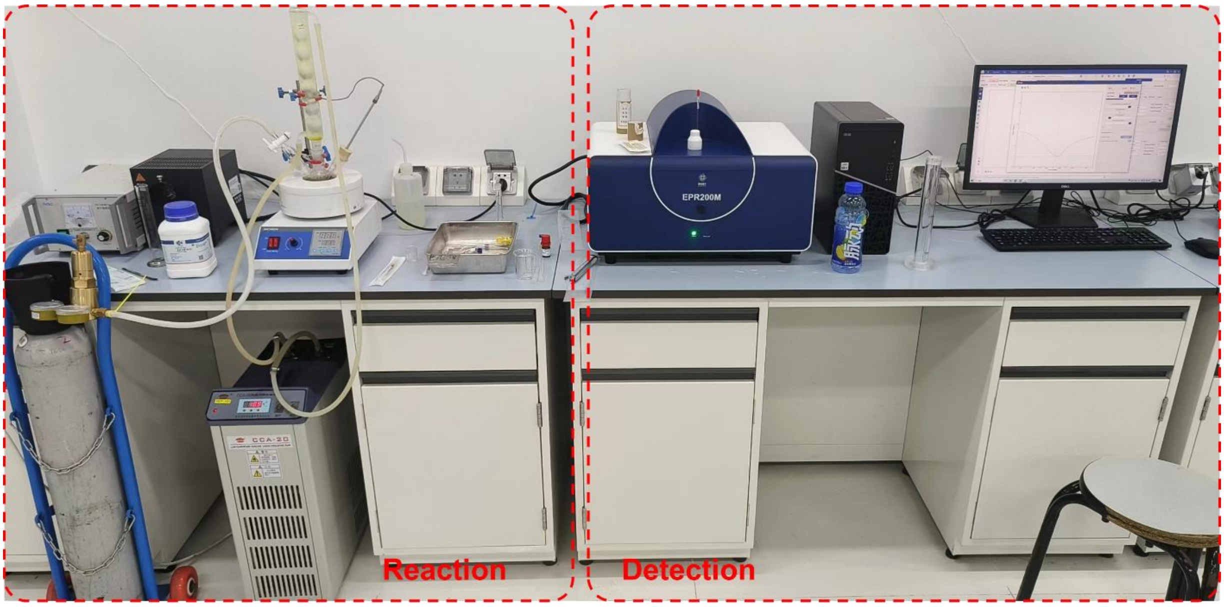

Supplementary Figure. 22. Ex-situ EPR testing process.

Detailed testing process of Ex-situ EPR: Firstly, add the reactants to a 50ml three necked flask and use a heating mantle for heating. During this process, pure oxygen is introduced into the reactor. When the corresponding reaction time is reached, quickly take out 1ml of the reaction solution and add 100μL of DMPO (5,5-Dimethyl-1-Pyrroline-N-oxide) to capture the hydroxyl radicals in the solution. Prepare liquid samples using capillary tubes to detect the intensity of hydroxyl radicals. The EPR spectra were collected between 3444 and 3544G in 100ms. The microwave frequency was 9.7GHz with a power of 0.2mW, and the field was modulated at 100kHz and with an amplitude of 5G.

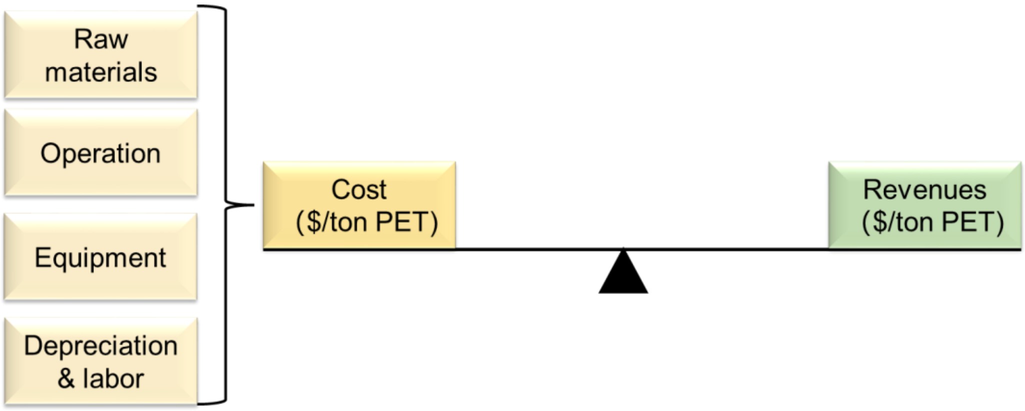

Supplementary Figure 23. The cost and income for calculating techno-economic analysis of thermal catalytic oxidation upcycling of PET.

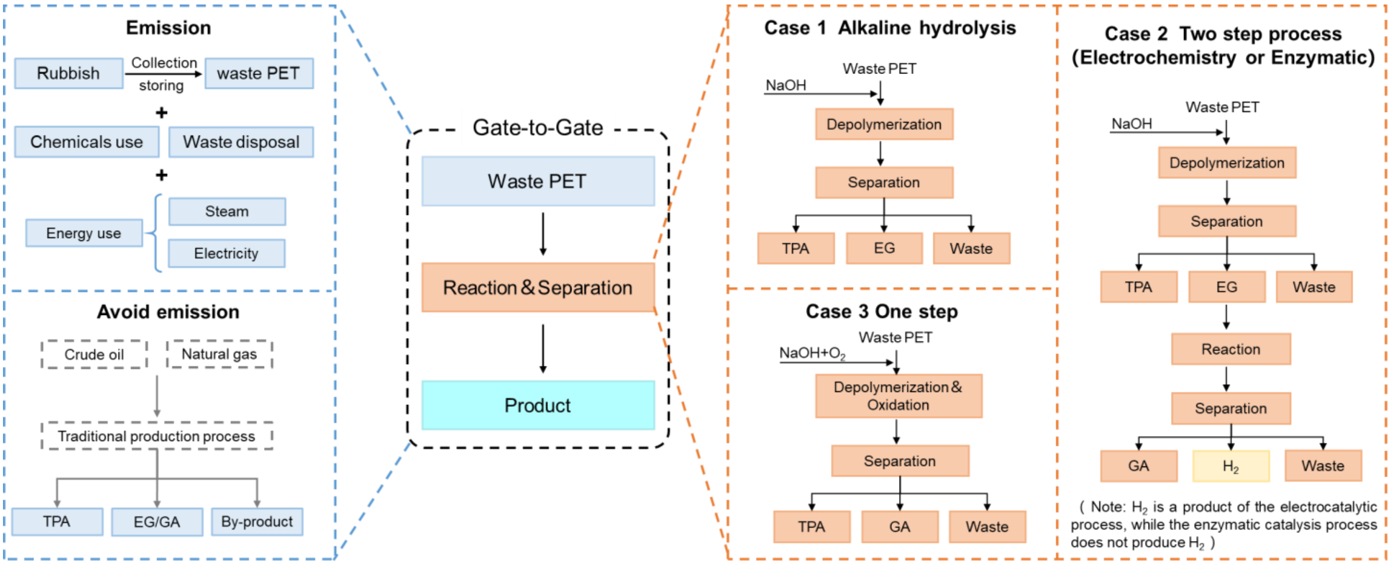

Supplementary Figure 24. LCA system boundary of chemical recycling waste PET to chemical products. The blue boxes represent the emissions generated and avoided during the production process. Different reaction routes are displayed within the orange boxes.

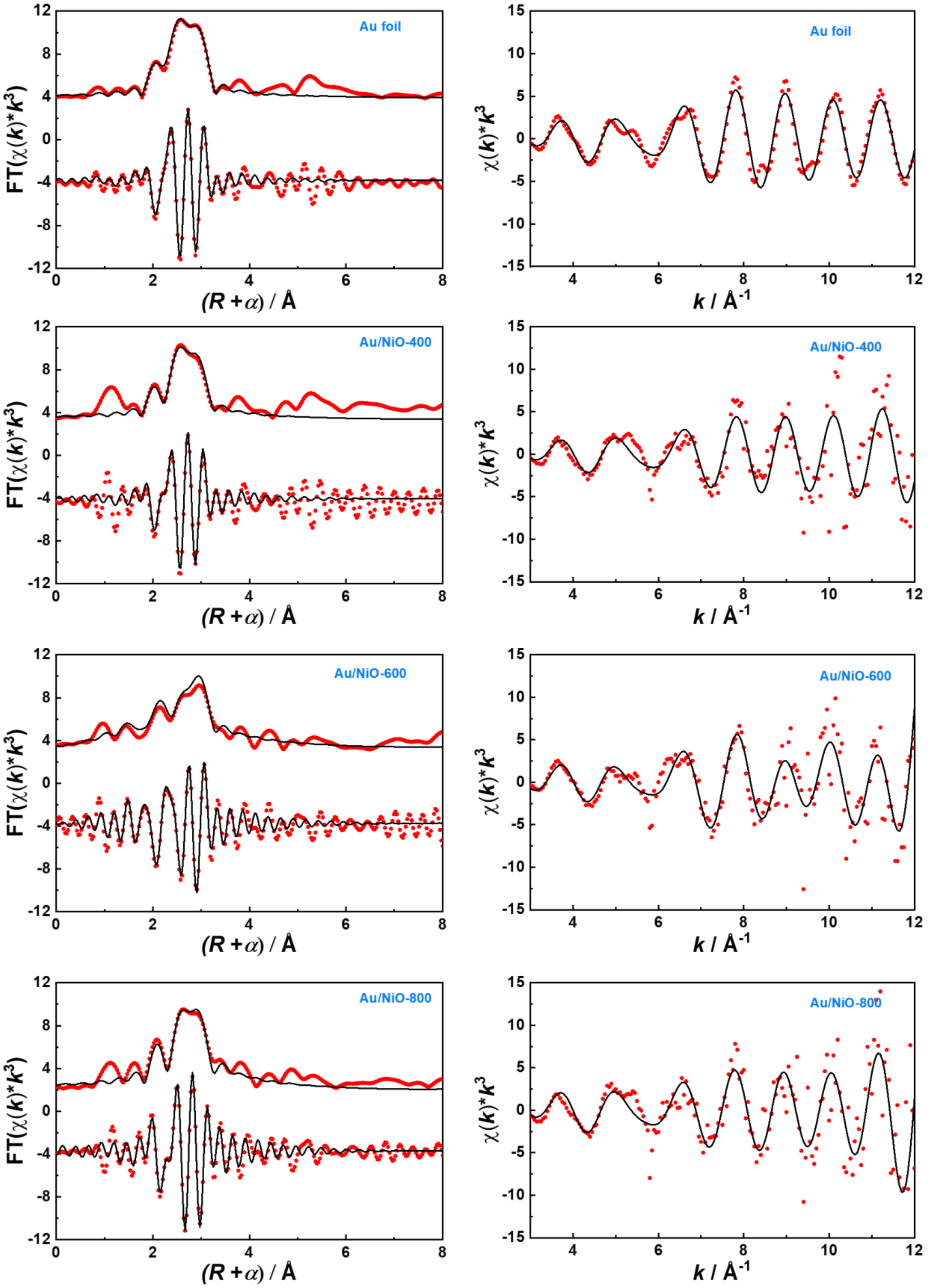

Supplementary Figure 25. K-edge EXAFS (points) and the curvefit (line) for Au foil, Au/NiO-400, Au/NiO-600, Au/NiO-800. (left: shown in R-space; right: shown in k*weighted k-space).

II. Supplementary Tables

Supplementary Table 1. Oxidation of PET over Au/NiO catalysts.

Catalyst	PET Conversion (%)	Yield (%)				EG oxidation rate (mmol/g/h)	C%
		TPA	EG	GA	Others		
**Blank**	100	99.08	94.63	0.45	3.84	-	98.12
**Au/NiO-C**	100	99.30	38.21	57.44	4.41	-	98.54
**[a]Blank**	64.25	63.52	62.52	0.57	0.43	-	97.53
**[a]Au/NiO-C**	70.28	68.32	34.25	31.44	2.63	-	97.17
**Au/Ni(OH)2**	100	99.12	15.28	33.13	50.74	5.65	98.21
**Au/NiO-250**	100	99.25	2.30	51.41	45.55	19.78	98.76
**Au/NiO-400**	100	99.64	7.85	80.28	11.51	47.74	98.91
**Au/NiO-600**	100	99.46	23.92	61.50	14.04	26.80	98.65
**Au/NiO-800**	100	99.21	44.55	43.92	10.74	15.93	98.43

Note:

1) C%:carbon balance, others: formic acide and CO_2.

$$\text{Carbon balance (\%)}=\frac{N_{TPA.yield}+N_{EG.yield}+N_{OA.yield}+N_{FA.yield}+N_{GA.yield}+N_{CO_2.yield}}{2\times N_{PET.reacted}}\times 100\% \quad (2\text{-}1)$$

TPA: Terephthalic acid; EG: Ethylene glycol; OA: Oxalic acid; FA: Formic acid; GA: Glycolic acid.

2) Reaction condition: 1g PET, 20ml H_2O, 0.8g NaOH, 0.1g catalyst, 1MPa O_2, 130°C, 3h. a: 1.5h.

3) Taking the Au/NiO-C catalyst as an example, the calculation process of thermal catalytic oxidation upcycling of PET to glycolic acid is as follows:

$$\text{PET conversion (\%)}=\frac{m_{PET.addition}-m_{PET.unreacted}}{m_{PET.addition}}\times 100\%=\frac{1-0}{1}\times 100\%=100\% \quad (2\text{-}2)$$

$$\text{TPA yield (\%)}=\frac{m_{TPA.yield}}{\frac{m_{PET.addition}}{192}\times 166}\times 100\%=\frac{0.8621}{\frac{1}{192}\times 166}\times 100\%=99.63\% \quad (2\text{-}3)$$

$$\text{GA yield (\%)}=\frac{N_{GA.yield}}{\frac{m_{PET.addition}}{192}}\times 100\%=\frac{0.299}{\frac{1}{192}}\times 100\%=57.54\% \quad (2\text{-}4)$$

$$\text{EG yield (\%)}=\frac{N_{EG.yield}}{\frac{m_{PET.addition}}{192}}\times 100\%=\frac{0.00199}{\frac{1}{192}}\times 100\%=38.21\% \quad (2\text{-}5)$$

$$\text{OA yield (\%)}=\frac{N_{OA.yield}}{\frac{m_{PET.addition}}{192}}\times 100\%=\frac{0}{\frac{1}{192}}\times 100\%=0\% \quad (2\text{-}6)$$

$$\text{Others yield (\%)=FA yield+}CO_2\text{ yield+OA yield=3.88\%} \quad (2\text{-}7)$$

$M_{(TPA)}$=166 g/mol, $M_{(PET)}$=192 $g\cdot mol^{-1}$

[The detailed parameters :1 g PET, 0.1 g catalyst, $m_{PET,\ unreacted}$=0g, $m_{TPA.yield}$=0.8621g]

$$\text{EG oxidation rate}=\frac{\frac{m_{TPA.yield}}{166}-N_{GA.yield}}{m_{catalyst}\cdot \text{Time}}=\frac{\frac{0.1271}{166}-0.00076}{0.1\times\frac{10}{60}}\times 1000=26.1(mmol\cdot h^{-1}\cdot g^{-1}{}_{catalyst}) \quad (2\text{-}8)$$

[The detailed parameters :1 g PET, 14.69 TPA yield, 8.52% GA yield, 10min , 0.1 g catalyst]

The filter cake resulting from the initial filtration comprises undepolymerized PET and catalyst. Notably, the mass of the Au/NiO catalyst remains unchanged post-reaction with water (1 MPa O_2, 130°C, 10 hours). By deducting the added catalyst mass from the cake mass, the residual PET mass after the reaction ($m_{PET,\ unreacted}$) is determined. To adjust the pH=3, 1 M HCl is added to the filtrate obtained from the primary filtration. Subsequent to this adjustment, a complete precipitation of terephthalic acid occurs, and its quantity is quantified through a subsequent filtration ($m_{TPA.yield}$). Through high-performance liquid chromatography analysis of the component concentration, the molar amount of the component can be ascertained based on the recorded filtrate volume ($N_{GA.yield}$, $N_{EG.yield}$, $N_{OA.yield}$). Utilizing the initial PET input and the relative molecular weight of the repeating units, the molar amount of links can be computed, which equates to the molar amount of TPA and ethylene glycol resulting from the full depolymerization of PET. However, due to incomplete PET depolymerization during the reaction, the molar amount of ethylene glycol matches that of terephthalic acid, falling short of the molar amount of the chain linked to the PET reaction. The oxidation reaction rate of ethylene glycol is evaluated when the PET depolymerization rate is below 20%, indicating the consumption rate of ethylene glycol during the initial reaction. All product yields are determined based on the theoretical EG/TPA monomer quantity in PET.

Supplementary Table 2. Physicochemical parameters of samples.

Sample	Au (wt%)[a]	Au Mean Size (nm)[b]	BET (m^2/g)
Au/Ni(OH)$_2$	0.52	4.5	38.08
Au//NiO-250	0.53	4.7	31.66
Au/NiO-400	0.53	4.6	25.37
Au/NiO-600	0.54	4.7	9.99
Au/NiO-800	0.52	5.0	1.56

a Determined by ICP-AES. b Determined by HRTEM.

Supplementary Table 3. Curvefit Parameters[a] for Au K-edge EXAFS fitting parameters for various samples.

Sample	Shell	S_0^2	σ^2/Å^2	d[b]/Å	R/Å	ΔE	CN
Au foil	Au-Au	0.90	0.007±0.001	2.82	2.859±0.004	4.33±0.40	11.3±0.61
Au/NiO-400	Au-Ni	0.85	0.003±0.001	2.85	2.86±0.054	3.50±1.13	0.7±0.44
	Au-Au	0.85	0.007±0.001	2.82	2.84±0.014		9.0±0.71
Au/NiO-600	Au-Ni	0.98	0.023±0.011	2.61	2.62±0.006	3.29	0.4±0.38
	Au-Au	0.98	0.008±0.001	2.82	2.85±0.014		9.9±1.10
Au/NiO-800	Au-Ni	1.00	0.01±0.023	2.85	2.87±0.024	4.03±2.31	0.1±0.35
	Au-Au	1.00	0.01±0.004	2.82	2.86±0.016		10.7±2.31

S_0^2: amplitude reduction factor; σ^2: Debye-Waller factor; d: set distance; R: fitted distance; CN: coordination number.

[a] Date ranges: 3.0 ≤k≤ 12.3 ,1.3≤R≤3.5 Ã. The number of variable parameters is 4、6、8、7， out of a total of 14.16、11.36、13.46、11.19 independent data points. R factor for these fits are 0.7 %、2.2%、2.4%、2.3%.

[b] The distances for Au-Au are from thecrystal structure of Au; the distance of Au-Ni is from thecrystal structure of Ni_3Au.

It was assumed that the Debye-Waller factor is the same for all the first-shell metal pairs (Au-Au and Au-Ni) to minimize the number of fitting parameters.

Supplementary Table 4. Ethanol aldehyde oxidation under the condition of adding quencher.

Number	Reaction conditions	GA Yield
1	Glycolaldehyde Dimer	12.31%
2	Glycolaldehyde Dimer<br>& Salicylic acid	5.18%
3	Glycolaldehyde Dimer<br>& Benzoquinone	11.98%

Reaction condition: 20ml 0.15M glycolaldehyde dimer, 0.8g NaOH, 0.1g Au/NiO-400 100ºC-10min

Supplementary Table 5. Technical and economic analysis of 100000 tons/year PET treatment plant based on traditional alkaline hydrolysis process.

	Project	Consumption of per Ton PET	Unit price ($/t)	Cost ($/ton PET)
**Cost**	**1. Raw materials**			**658**
	Waste PET	1	390	390
	NaOH	0.6	420	252
	HCl	0.8	20	16
	**2. Operation**			**194**
	Water	300	0.22	66
	Electricity	200	0.1	20
	Steam	4.5	24	108
	**3. Equipment**			**20.5**
	**4. Depreciation and labor**			**19.5**
	**Sum**			**892**
**Revenues**	**Product revenue**	**Production (t)**	**Unit price ($/t)**	**Revenue ($/ton PET)**
	TPA	0.84	1260	1058.4
	EG	0.28	756	211.68
	NaCl	0.8	90	72
	**Sum**			**1342.08**
**Gross profit**	**446.08 $/t PET**			

TPA: 1,4-dicarboxybenzene; EG: Ethylene glycol

Supplementary Table 6. Technical and economic analysis of 100000 tons/year PET treatment plant based on the one-pot conversion.

	Project	Consumption of per Ton PET	Unit price ($/t)	Cost ($/ton PET)
Cost	**1. Raw materials**			**802.75**
	Waste PET	1	390	390
	O_2	0.27	100	27
	NaOH	0.6	420	252
	HCl	0.8	20	16
	Ethanol	0.15	785	117.75
	**2. Operation**			**170**
	Water	200	0.22	44
	Electricity	300	0.1	30
	Steam	4	24	96
	**3. Equipment**			**50**
	**4. Depreciation and labor**			**22**
	**Sum**			**1044.75**
Revenues	**Product revenue**	**Production (t)**	**Unit price ($/t)**	**Revenue ($/ton PET)**
	TPA	0.82	1260	1033.2
	GA	0.3	4879	1463.7
	NaCl	0.63	90	56.7
	**Sum**			**2553.6**
**Gross profit**	**1508.85 $/t PET**			

TPA: 1,4-dicarboxybenzene; GA: Glycolic acid

Supplementary Table 7. Technical and economic analysis of 100000 tons/year PET treatment plant based on the Electrocatalytic route.

	Project	Consumption of per Ton PET	Unit price ($/t)	Cost ($/ton PET)
**Cost**	**1. Raw materials**			**903**
	Waste PET	1	390	390
	NaOH	0.8	420	336
	HCl	1	20	20
	Ethanol	0.2	785	157
	**2. Operation**			**188**
	Water	300	0.22	66
	Electricity	600	0.1	50
	Steam	3	24	72
	**3. Equipment**			300
	**4. Depreciation and labor**			85.5
	**Sum**			**1476.5**
	**Product revenue**	**Production (t)**	**Unit price ($/t)**	**Revenue ($/ton PET)**
**Revenues**	TPA	0.82	1260	1033.2
	GA	0.24	4879	1170.96
	H_2	0.0197	2790	54.963
	NaCl	1.08	90	97.2
	**Sum**			**2356.32**
**Gross profit**	**879.82 $/t PET**			

TPA: 1,4-dicarboxybenzene; GA: Glycolic acid

Supplementary Table 8. Technical and economic analysis of 100000 tons/year PET treatment plant based on bioconversion.

	Project	Consumption of per Ton PET	Unit price ($/t)	Cost ($/ton PET)
**Cost**	**1. Raw materials**			**1020**
	Waste PET	1	390	390
	Medium	0.15	4200	630
	**2. Operation**			**84.8**
	Water	120	0.22	26.4
	Electricity	200	0.1	20
	Steam	1.6	24	38.4
	**3. Equipment**			**153**
	**4. Depreciation and labor**			**50**
	**Sum**			**1307.8**
**Revenues**	**Product revenue**	**Production (t)**	**Unit price ($/t)**	**Revenue ($/ton PET)**
	TPA	0.67	1260	8844.2
	GA	0.21	4879	1024.59
	**Sum**			**1868.79**
**Gross profit**	**560.99 $/t TPA**			

TPA: 1,4-dicarboxybenzene; GA: Glycolic acid

Supplementary Table 9. Thermal catalytic oxidation of PET to glycolic acid compared with electrocatalysis and bioconversion.

	**Thermal conversion**	**Electrocatalysis**	**Bioconversion**
Operation steps	1	2	2
Glycolate yield	75.7%	Max.58.5%	40.70%
reaction time	3h	3.5+10h	3.5+20h
Oxidant	Oxygen	H_2O	H_2O
By-product	Oxalic acid; Formate	Formate	complex
G-facter<br>(G-facter: Glycolate tons/PET tons)	0.3	0.23	0.11

Supplementary Table 10. Comparison of Carbon Dioxide Emissions from Different Recycling Routes.

Number	Recycling method	Product	NREU (MJ/kg plastic)	GWP (kg CO_2-eq/kg plastic)
1	Mechanical recycling	Recycled plastic	--	-1.2
2	Mechanical recycling	Plastic bottle	--	-1.7
	Burning	Electricity	--	1.4
3	Landfilling	Solid waste	5.65	10.5
	Incineration	Energy	-10.8	4.94
4	Methanolysis	DMT+EG	--	-1.88
5	Alkaline hydrolysis	TPA+EG	--	-1.17
6	Acetolysis	TPA+EDGA	70	2.19
7	Glycolysis	BHET	66	3.66
8	Pyrolysis	TEA	3.2	0.40
		BTX	-18.1	1.88
9	This work	TPA+GA	-37.09	-2.25

[1] Waste Manag. Res. 2009, 27 (8), 763−772.
[2] Resour. Conserv. Recycl. 2010, 54 (12), 1241−1249.
[3] J. Clean. Prod. 2019, 211, 1268−1283.
[4] ACS Sustain Chem. Eng. 2024, 12, 4114−412.
[5] Chem. Eng. J. 2023, 470, 1385-8947.
[6] Nat Commun. 2023,14, 3249
[7] Resour, Conserv. Recycl. 2010, 55 (1), 34-52
[8] Energy Environ. Sci. 2023, 16, 3638–3653

III. Supplementary Methods

1.Materials

Polyethylene terephthalate (PET, 100 mesh)

Polyethylene naphthalate two formic acid glycol este (PEN, TW8065s)

Poly(ethylene adipate) (PEA, ~1000, Aladdin)

Polyethylene 2,5-furandicarboxylate (PFE, 0.76dl/g)

NaOH (AR, Sinopharm Chemical Reagent Co., Ltd)

Ethylene glycol ($C_2H_6O_2$, AR, Sinopharm Chemical Reagent Co., Ltd)

Glycolic acid ($C_2H_4O_3$, Sinopharm Chemical Reagent Co., Ltd)

Oxalic acid ($C_2H_2O_4\cdot 2H_2O$, AR, Sinopharm Chemical Reagent Co., Ltd)

Formic acid (CH_2O_2, ≥99.5%, Sinopharm Chemical Reagent Co., Ltd)

Terephthalic acid ($C_8H_6O_4$, 99%, Sinopharm Chemical Reagent Co., Ltd)

Disodium Terephthalate ($C_8H_4Na_2O_4$, 99%. Sinopharm Chemical Reagent Co., Ltd)

Hydrochloric acid (HCl, 37 wt%, Sinopharm Chemical Reagent Co., Ltd)

Nickel(II) oxide (NiO, 100GR Sinopharm Chemical Reagent Co., Ltd)

Nickel(II) nitrate hexahydrate ($Ni(NO_3)_2\cdot 6H_2O$, AR, Sinopharm Chemical Reagent Co., Ltd)

Ammonia water (NH_4OH, GR, Sinopharm Chemical Reagent Co., Ltd)

Tetrachloroauric(III) acid tetrahydrate ($HAuCl_4\cdot 4H_2O$, AR Sinopharm Chemical Reagent Co., Ltd)

2.Reaction kinetics calculation

I. Elimination of the diffusion limitations

The Au/NiO-400 catalyst was used to study the internal and external mass transfer. It is ensured that all the catalysts were evaluated with negligible mass transfer limitations.

(1) Effect of external diffusion on EG oxidation

[Reaction conditions: 80℃, 1MPa O_2, 20ml Reaction solution (0.1M EG and 0.1M TPA-Na), 0.4g NaOH, 0.1 g Cat.]

(a) At 500 RPM, (10 min) $R_{oxidation} = 0.365\ kmol\cdot m^{-3}\cdot h^{-1}$;

(b) At 800 RPM, (10 min) $R_{oxidation} = 0.\ 359\ kmol\cdot m^{-3}\cdot h^{-1}$;

(c) At 1000 RPM, (10 min) $R_{oxidation} = 0.368\ kmol\cdot m^{-3}\cdot h^{-1}$;

(2) Interphase Mass Transfer Limitation for Oxygen

(a) Gas-Liquid Mass Transfer Limitation: (Three phase catalytic reactors, Ramachandran & Chaudhari, 1983; J. Chem Eng Data. 1984, 29, 286-287; Chem. Eng. Process, 2004, 43, 823-830; J. Catal. 2016, 337, 272-283; J. Catal. 2008, 257, 1-4)

$$\frac{R_{oxidation} d_{bubble}}{6 \cdot \varepsilon \cdot k_{g\text{-}l} \cdot C_{O_2,b}} = \frac{0.359(kmol \cdot m^{-3} \cdot h^{-1}) \cdot 0.000002(m)}{6 \cdot 0.09 \cdot 1.44(m \cdot h^{-1}) \cdot 0.01(kmol \cdot m^{-3})} = 9.2 \times 10^{-5} < 0.1 \quad (3\text{-}1)$$

(b) Liquid-Solid Mass Transfer Limitation: (J. Catal. 2016, 337, 272-283; Three phase catalytic reactors, Ramachandran & Chaudhari, 1983)

$$\frac{R_{oxidation} \cdot \rho_p \cdot d_p}{6 \cdot w_{cat} \cdot k_{l\text{-}s} \cdot C^*_{O_2,b}} = \frac{0.359(kmol \cdot m^{-3} \cdot h^{-1}) \cdot 2000(kg \cdot m^{-3}) \cdot 10^{-7}(m)}{6 \cdot 6.7(kg \cdot m^{-3}) \cdot 72.1(m \cdot h^{-1}) \cdot 0.01(kmol \cdot m^{-3})} = 2.5 \times 10^{-6} < 0.1 \quad (3\text{-}2)$$

(c) Internal Diffusion: (J. Catal. 2016, 337, 272-283; Three phase catalytic reactors, Ramachandran & Chaudhari,

1983; Perry's Handbook: Table 5-16

$$\frac{R_{oxidation} \cdot \rho_p \cdot d_p^2}{6 \cdot w_{cat} \cdot D_e \cdot C^*_{O_2}} = \frac{0.359(kmol \cdot m^{-3} \cdot h^{-1}) \cdot 2000(kg \cdot m^{-3}) \cdot (10^{-7}(m))^2}{6 \cdot 6.7(kg \cdot m^{-3}) \cdot 0.094(m^2 \cdot h^{-1}) \cdot 0.01(kmol \cdot m^{-3})} = 2.9 \times 10^{-10} < 0.1 \quad (3\text{-}3)$$

(3) Interphase Mass Transfer Limitation for Glycerol

(a) Liquid-Solid Transfer Limitation: (J. Catal. 2016, 337, 272-283; AIChE J. 1980, 26, 177-201; Three phase catalytic reactors, Ramachandran & Chaudhari, 1983)

$$\frac{R_{oxidation} \cdot \rho_p \cdot d_p}{6 \cdot w_{cat} \cdot k_{l\text{-}s} \cdot C_{eg}} = \frac{0.359(kmol \cdot m^{-3} \cdot h^{-1}) \cdot 2000(kg \cdot m^{-3}) \cdot 10^{-7}(m)}{6 \cdot 6.7(kg \cdot m^{-3}) \cdot 0.6(m \cdot h^{-1}) \cdot 0.1(kmol \cdot m^{-3})} = 3.0 \times 10^{-5} < 0.1 \quad (3\text{-}4)$$

(b) Intraparticle Transfer Limitation: (J. Catal. 2016, 337, 272-283; AIChE J. 1980, 26, 177-201; Three phase catalytic reactors, Ramachandran & Chaudhari, 1983)

$$\frac{d_p}{6}\left[\frac{(m+1) \cdot R_{oxidation} \cdot \rho_p}{2 \cdot w_{cat} \cdot D_e \cdot C_{eg}}\right]^{0.5} = \frac{10^{-7}(m)}{6}\left[\frac{0.359(kmol \cdot m^{-3} \cdot h^{-1}) \cdot 2000(kg \cdot m^{-3})}{2 \cdot 6.7(kg \cdot m^{-3}) \cdot 0.036(m^2 \cdot h^{-1}) \cdot 0.1(kmol \cdot m^{-3})}\right]^{0.5} = 2.0 \times 10^{-6} < 0.2 \quad (3\text{-}5)$$

II. Calculation of apparent reaction kinetics

The reaction order of EG in different reaction systems on Au/NiO-X catalysts was investigated by using power function reaction kinetics equation. The equation can be expressed as :

$$r = -\frac{dC_0}{dt} = A \exp\left(\frac{-Ea}{RT}\right) C_{EG}^a P_{O_2}^b \quad (3\text{-}6)$$

a and b are the corresponding reaction order. r, T, A, R and Ea are the initial reaction rate of glycerol ($kmol \cdot L^{-1} \cdot h^{-1}$), reaction temperature (K), the pre-exponential factor, ideal gas constant (8.314×10^{-3} $kJ \cdot mol^{-1} \cdot K^{-1}$) and activation energy ($kJ \cdot mol^{-1}$).

3.Life cycle assessment

I. Goal and scope definition

The aim of this study is to evaluate the environmental impact of four PET recycling routes. The system boundary of LCA is from gate to gate, including the production of input resources (i.e., NaOH, HCl, and O_2), the treatment of PET and waste disposal. To simplify the research, the cut-off method was used for the treatment of PET. Waste PET does not bear the environmental burden from the prior life, only examines the process from PET to the product. The functional unit is 1kg waste PET fragments. Calculating the consumption of production processes and greenhouse gas emissions, and recovering TPA/EG/GA is considered to avoid the consumption of fossil fuels and greenhouse gas emissions through traditional pathways to produce equal quantities of products. As shown in Supplementary Fig. 15, LCA follows the ISO standard series 14040.

II. Life cycle inventory and impact assessment

The lifecycle inventory of PET recycling process was modeled using OpenLCA v 2.1.1 software. Table 1 provides the complete mass balance and energy use for calculation, with data sourced from relevant literature. The background processes (NaOH, HCl, electricity, tap water, etc.) were modeled using the Ecoinvent v3.9.1 database (table 2). The data is mainly based on European datasets, and other regional datasets may be used if necessary. NREU uses CML v4.8 2016 for calculation, while GWP uses IPCC 2021 for calculation, with GWP-100 as the evaluation indicator. The calculation results are presented in Table 3, with the symbol (-) indicating the impact on environmental reduction.

III. Sensitivity analysis

By conducting sensitivity analysis on the model, the sensitivity of the model to parameter uncertainty can be obtained. The controlled variable method was used to investigate the degree of influence of H_2O, O_2, NaOH, and HCl on the results. Change by 10% each time and calculate the sensitivity ratio (SR) of each parameter. Table 4 shows the results of sensitivity analysis in the one-step oxidation model, and the input amount of materials has no significant impact on the LCA results. The SR values of all parameters are less than 1, indicating that the LCA model is robust to changes in input parameters.

$$SR=\frac{\frac{\Delta results}{Inittial\ results}}{\frac{\Delta parameter}{Inittial\ parameter}} \quad (3\text{-}7)$$

Table 1: input-output mass balance and energy consumption of recycling PET to chemicals

Process	Input			Output	
		Matter	Mass(Kg)	Matter	Mass(Kg)
Alkaline hydrolysis	Material	PET	1	TPA	0.84
		H_2O	30	EG	0.28
		NaOH	0.6	NaCl	0.77
		HCl	0.8	Waste	31.28
	Energy	Electricity (Kwh)	0.3		
		Heat (MJ)	0.5		
One step	Material	PET	1	TPA	0.82
		H_2O	20	GA	0.3
		NaOH	0.6	NaCl	0.71
		HCl	0.8	Alcohol	0.26
		Alcohol	0.1	Waste	21.78
		O_2	0.4		
	Energy	Electricity (Kwh)	0.26		
		Heat (MJ)	0.66		
Electrochemistry	Material	PET	1	TPA	0.85
		H_2O	30	GA	0.24
		NaOH	0.8	H_2	0.02
		HCl	1	NaCl	1.08
				Waste	31.6932
	Energy	Electricity (Kwh)	0.79		
		Heat (MJ)	0.34		
Enzymatic	Material	PET	1	TPA	0.67
		H_2O	12	GA	0.21
		Medium	0.15	Waste	12.27
	Energy	Electricity (Kwh)	0.47		
		Heat (MJ)	0.41		

Table 2: GWP and NREU values of each raw material used in the PET chemical recovery process in OpenLCA

	NREU		GWP	
Subprocess	Value	Unit	Value	Unit
PET-Sorting	3.71354	MJ/Kg	0.26802	Kg CO_2-eq/Kg
H_2O	0.00302	MJ/Kg	0.00026	Kg CO_2-eq/Kg
NaOH	9.83737	MJ/Kg	0.89524	Kg CO_2-eq/Kg
HCl	11.30373	MJ/Kg	0.91757	Kg CO_2-eq/Kg
O_2	0.16298	MJ/Kg	0.0412	Kg CO_2-eq/Kg
TPA	38.09298	MJ/Kg	2.95404	Kg CO_2-eq/Kg
EG	35.61372	MJ/Kg	1.56682	Kg CO_2-eq/Kg
GA	86.5438	MJ/Kg	4.73075	Kg CO_2-eq/Kg
H_2	66.84983	MJ/Kg	2.73778	Kg CO_2-eq/Kg
Waste	0.01137	MJ/Kg	0.00144	Kg CO_2-eq/Kg
NaCl	0.2513	MJ/Kg	0.15085	Kg CO_2-eq/Kg
Medium	44.3671	MJ/Kg	3.71561	Kg CO_2-eq/Kg
Electricity (Kwh)	1.10031	MJ/Kwh	0.11329	Kg CO_2-eq/Kg
Heat (MJ)	1.46801	MJ/MJ	0.10339	Kg CO_2-eq/Kg

Table 3: The NREU and GWP of recycling 1 kg PET to produce chemicals and potentially avoiding impact

	Process	Alkaline hydrolysis	One step	Electrochemistry	Enzymatic
NREU (MJ)	Emission	20.1694	20.2867	24.7066	10.5443
	Avoid	42.1332	57.3577	54.5440	43.6964
	Total	-21.9638	-37.0709	-29.8374	-33.1521
GWP (Kg CO_2-eq)	Emission	1.6777	1.6898	2.0798	0.9417
	Avoid	3.0181	3.9365	3.8552	2.9726
	Total	-1.3404	-2.2467	-1.7753	-2.0308

Table 4: Sensitivity ratio with increase in the input flows by 10%

	Sensitivity ratio NREU (MJ)	Sensitivity ratio GWP (Kg CO_2-eq)
H_2O	0.03	0.01
NaOH	0.21	0.17
HCl	0.19	0.25
O_2	0.05	0.09
